# Strokes and Diagnostic Dilemmas: Non-bacterial Thrombotic Endocarditis Mimicking a Flail Mitral Valve

**DOI:** 10.7759/cureus.87380

**Published:** 2025-07-06

**Authors:** Ahmed K Mahmoud, Reza Arsanjani, Chadi Ayoub

**Affiliations:** 1 Cardiovascular Medicine Department, Mayo Clinic, Phoenix, USA

**Keywords:** flail mitral valve, non-bacterial thrombotic endocarditis, therapeutic anticoagulation, transesophageal echocardiography (tee), transthoracic echocardiography (tte)

## Abstract

Non-bacterial thrombotic endocarditis (NBTE) is a rare condition characterized by sterile vegetations on cardiac valves, commonly associated with malignancy or autoimmune disorders. These vegetations pose a significant risk of embolization, leading to adverse complications such as ischemic stroke. We present a case of a 60-year-old female initially suspected of having a brain tumor, later diagnosed with multifocal strokes due to NBTE. Initial interpretation of a flail mitral valve was subsequently revised to NBTE on further assessment, but this resulted in a delay of anticoagulation initiation. This case highlights the importance of early recognition and multidisciplinary management of NBTE to prevent recurrent embolic events, highlighting the diagnostic challenges and the role of anticoagulation in its treatment.

## Introduction

Non-bacterial thrombotic endocarditis (NBTE), previously referred to as Libman-Sacks endocarditis, is an uncommon clinical entity defined by the formation of sterile, fibrin- and platelet-rich vegetations on cardiac valve leaflets without underlying infection [1]. The pathogenesis of NBTE is driven by a combination of endothelial injury, immune complex deposition, and hypercoagulable states, particularly in the context of underlying malignancy, systemic lupus erythematosus (SLE), and other autoimmune conditions, or antiphospholipid syndrome [2,3]. The estimated incidence in autopsy series ranges from 0.3% to 1.2% overall, with higher frequencies up to 4% in patients with advanced malignancy and as high as 32% in those with cancer and concomitant ischemic stroke [1,2]. Mucinous adenocarcinomas (notably of the pancreas and lung) are particularly associated with NBTE [2,3]. NBTE most commonly affects individuals in the fourth to eighth decades of life [4,5].

Clinically, NBTE often presents with systemic embolic phenomena rather than overt cardiac symptoms. Embolic complications are common, with stroke occurring in 33-57% of cases, and other organ involvement including the spleen, kidney, coronary, and peripheral arteries. Heart failure due to valvular dysfunction is less frequent but may be observed in advanced cases where the mitral leaflets are damaged and severe mitral regurgitation ensues [1,3]. The differential diagnosis for NBTE is broad and includes infective endocarditis, degenerative valvular disease, rheumatic heart disease, and other non-infective etiologies. Differentiation from infective endocarditis is particularly critical and relies on the absence of bacteremia, negative blood cultures, inflammatory markers, and fulfillment of the modified Duke’s criteria [1,2]. Characteristic imaging findings on echocardiography may also help in differentiating these entities.

Echocardiography, both transthoracic (TTE) and transesophageal (TEE), is central to the detection of valvular vegetations, but NBTE vegetations are often smaller, more friable, and less likely to cause destruction compared with those seen in infective endocarditis [5]. The classic findings are vegetations on the mitral leaflet tips, which are characteristic of the inflammatory process and termed ‘kissing lesions’. Laboratory investigations often show evidence of an underlying hypercoagulable state, and autoimmune markers (such as antiphospholipid antibodies) may assist in establishing contributory diagnoses [2].

Definitive management of NBTE centers on prompt and sustained therapeutic anticoagulation, usually with unfractionated or low-molecular-weight heparin, combined with directed treatment of the underlying disease process [2,3]. Warfarin is generally used, and heparin is then discontinued once the international normalized ratio (INR) is therapeutic; however, there is limited data for direct oral anticoagulants in this setting [1,3]. Surgical intervention, such as valve replacement, is reserved for patients who develop severe valvular dysfunction or intractable embolic events despite medical therapy [2,3].

We report a case of a patient with multifocal strokes, which was eventually found to be secondary to embolization from NBTE. The vegetations were initially interpreted as a flail mitral valve based on leaflet motion and morphology on TEE, which mimicked the kissing lesions of the mitral valve that were better appreciated on subsequent TTE, leading to a delay in critically needed anticoagulant therapy.

## Case presentation

**Figure 1:**
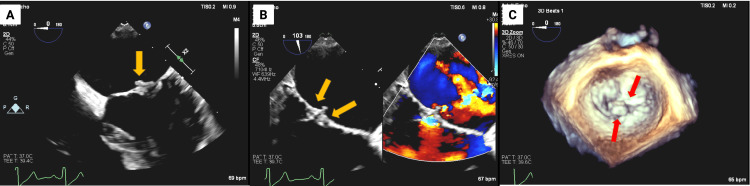
Transesophageal echocardiography (TEE) images. The TEE was the initial cardiac imaging test performed after the diagnosis of multifocal strokes was made. Panel A demonstrates the appearance that led to the initial reporting of flail mitral valve appearance (yellow arrow). Thickened mitral valve leaflet tips (both anterior and posterior leaflets), with kissing lesion appearance (yellow arrows), are shown in panel B, raising suspicion for NBTE and inflammatory etiology. Panel C shows a 3D reconstruction of the mitral valve, with thickening of both mitral leaflet tips (red arrows). NBTE: non-bacterial thrombotic endocarditis

A 60-year-old female patient presented with an out-of-hospital episode of right lower facial paresthesias with numbness and associated slurred speech, prompting brain magnetic resonance imaging (MRI), which raised suspicion for an intracranial neoplasm due to multifocal enhancing lesions. However, further MRI review confirmed strokes, represented by multiple cortical infarcts in the left frontal and right parietal lobes, raising concern for a cardioembolic source. TEE was accordingly performed and initially thought to be a flail mitral valve with mild-to-moderate mitral regurgitation (Figure 1). This interpretation was based on mitral leaflet exaggerated motion, which mimicked the flail leaflet appearance.

**Figure 2:**
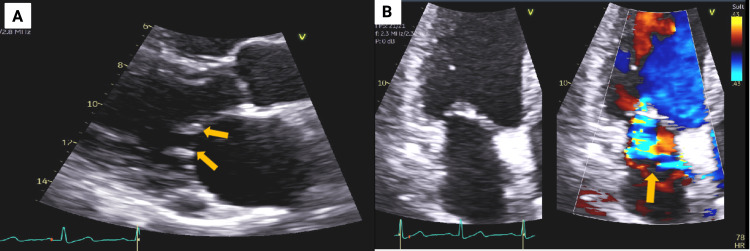
Transthoracic echocardiogram (TTE) images. The TTE was ordered after the diagnostic challenges with the TEE. Panel A demonstrates a parasternal long-axis view showing large irregular echo densities on both mitral leaflet tips, consistent with ‘kissing lesion’ (yellow arrows). Panel B shows color Doppler imaging across the mitral valve, with mild to moderate mitral regurgitation (yellow arrow).

Upon re-evaluation and with the performance of transthoracic echocardiography (TTE), thickening of the mitral leaflet tips and opposing vegetation, “kissing lesions” on the anterior and posterior leaflets, were better appreciated, which are suggestive of an inflammatory etiology (Figure 2). Laboratory testing revealed positive lupus anticoagulant and heterozygous factor 5 Leiden mutation, which were ordered subsequently after review of cardiac imaging, revised the impression to vegetations on the mitral leaflets. These findings were consistent with NBTE, which mimicked the appearance of mitral flail and resulted in the failure of prompt commencement of anticoagulation. 

The patient was accordingly admitted for heparin bridging and commencement of warfarin therapy, and a comprehensive hematologic evaluation was undertaken to exclude malignancy in the setting of lymph nodes identified on positron emission tomography scan (PET). After initiation of therapeutic anticoagulation, the patient experienced no further embolic events and had gradual neurologic improvement. Furthermore, the oncology workup did not reveal any evidence of an underlying malignancy, and the final diagnosis was NBTE in the setting of antiphospholipid syndrome.

## Discussion

This case highlights the critical importance of recognition and early diagnosis of NBTE to avoid treatment delays and decrease the risk of recurrent embolic events. This case demonstrates a diagnostic dilemma: NBTE was not initially considered due to overreliance on TEE findings suggestive of structural valve disease. The lack of infective signs and the presence of thrombophilia were not integrated early into the diagnostic reasoning. NBTE is a rare but serious complication often associated with underlying malignancy, autoimmune diseases, or hypercoagulable states [2]. It is characterized by the deposition of sterile thrombi on cardiac valves, most commonly the mitral and aortic valves, leading to embolic phenomena that can result in cerebrovascular events. In this case report, identification of NBTE was essential, as delayed diagnosis could have resulted in additional ischemic strokes or systemic embolization [2].

Without appropriate anticoagulation, this patient would have remained at high risk for further thromboembolic events. Although there was an initial concern regarding surgical intervention for the reported flail mitral leaflet, subsequent review of the TEE images and evaluation suggested NBTE. NBTE can mimic flail mitral leaflet appearance, but unlike flail, NBTE vegetations often appear as irregular, non-mobile masses at the leaflet tips without true prolapse or ruptured chordae. Therapeutic anticoagulation remains a cornerstone of management in NBTE; however, no formal guidelines exist for its optimal treatment. Warfarin is traditionally preferred over direct oral anticoagulants (DOACs) due to the limited data on DOAC efficacy in this condition [6]. Some reports suggest that heparin-based therapy may be more effective in preventing recurrent embolization, particularly in patients with active malignancy [6].

The presence of axillary lymphadenopathy on a PET scan raises suspicion for an underlying malignancy, a known predisposing factor for NBTE. Malignancy-associated NBTE is often seen in advanced stages of cancer, particularly in adenocarcinomas, and warrants a thorough workup [4]. Given this patient's clinical presentation, further evaluation, including biopsy and additional oncologic assessment, was undertaken to rule out an occult malignancy; such a rule-out would be recommended in similar presentations of cases [7].

Multidisciplinary collaboration is crucial in managing NBTE, as it requires coordinated care. Neurology guided stroke evaluation and workup, cardiology managed imaging and anticoagulation decisions, hematology coordinated thrombophilia testing and hypercoagulability management, and oncology directed further malignancy screening. The multidisciplinary approach ensures comprehensive evaluation of underlying etiologies, appropriate anticoagulation strategies, and close monitoring for treatment response or complications [8].

## Conclusions

This case highlights the importance of early suspicion for NBTE in patients with prior stroke who present with echocardiographic findings such as leaflet tip thickening or irregular vegetations. In such instances, it is crucial to assess for an underlying hypercoagulable state. Review of cardiac imaging is critical and may require careful re-evaluation, taking into account the clinical context to avoid misdiagnosis. Early diagnosis, appropriate anticoagulation, and a multidisciplinary approach remain essential for optimizing patient outcomes.
